# Supplementary material for: Genetic and pharmacological interrogation of cancer vulnerability using a multiplexed cell line screening platform
Source: Commun Biol. 2021 Jul 2;4:834. doi: 10.1038/s42003-021-02352-2 (PMC8253734; doi:10.1038/s42003-021-02352-2)
Supplement: Supplementary file 2 — Supplementary Information [file 42003_2021_2352_MOESM2_ESM.pdf]

Genetic and Pharmacological Interrogation of Cancer Vulnerability Using a Multiplexed Cell Line Screening Platform

Yifeng Xia[1,*], Xiaodong Ji[1,*], In Sock Jang[1], Christine Surka[1], Christy Hsu[1], Kai Wang[1], Mark Rolfe[1], Neil Bence[1], Gang Lu[1]

[1]Bristol Myers Squibb, San Diego, CA, USA

Supplementary Methods

I. Barcoding Cancer Cell Lines

A. Lentiviral preparation.

- Transfect Lenti-X 293T (Clontech) cells in 6-well plate with Cas9 plasmid containing the barcode.
- Collect viruses after 48h. The typical viral titer should be around $2x10^6$/ml.
- Viruses are used for infection freshly (within 7 days in 4°C), or can be frozen in -80°C.

B. Cell line Cas9-enabling and barcoding.

- Thaw cells to be engineered.
- Day 0: Plate $2.5x10^5$ cells in 12-well plates (2 wells per cell line, 1 will be infected and 1 will not be infected and serve as control). Add 1 ml of lentiviral stock and 4 µg/ml Polybrene for infection.
- Day 1: Change to fresh medium.
- Day 2: Split the cells and seed to 6-well plate. Start Blasticidin selection (typically 20 µg/ml, up to 40 µg/ml for some cell lines, to completely kill cells in control well) to enrich infected cells.
- Day x: Expand cells to 10 cm dish under Blasticidin selection.

C. Cas9 activity test.

- Prepare reporter virus stock. The typical viral titer should be around $5x10^6$/ml.
- Day 0: Plate $2.5x10^5$ cells in 12-well plates (2 wells per cell line, 1 with parental cells and 1 with Cas9 enabled cells). Add 0.05 ml of reporter viral stock for infection.
- Day 1: Change to fresh medium.
- Day 3: Split the cells, leave ¼ of the cells in the well. The rest of the cells are used for cytometry flow assay to determine percentage of RFP and GFP positive cells.
- Day 6: Trypsinize the cells and repeat the cytometry flow assay as on Day 3.
- Analysis. The parental cells should be either RFP+GFP+ or RFP-GFP-. (RFP+GFP+)% on Day 3 is used to estimate cell infectability. The Cas9 enabled cells should be RFP+GFP+, RFP-GFP+, or RFP-GFP-. Cas9 efficiency is determined by: (GFP-RFP+)%/(all RFP+)%

II. Mixing Cancer Cell Lines

- After Cas9 efficiency test, the Cas9 enabled and barcoded cells are expanded and frozen down individually as stocks.
- For convenience, ~20 individual Cas9 enabled and barcoded cell lines (typically from same tumor lineage) are further subpooled and frozen down (1.5 $x10^5$ cells per line/vial).

III. Compound screen (workflow shown in Supplementary Figure 7)

- Day -1: Thaw cells from frozen subpools. Seed 5 $x10^5$ cells/well with 3 ml medium in 6-well plate. **Cell number estimation: total 400 lines, ~1250 cells/line.**
- Day 0: Add compounds to wells at the final concentrations of 0, 0.01 µM, 0.1 µM, 1 µM, 10 µM.
- Day 3: Split cells from each well into two 100 mm dishes: 1/5 cells for day 10 sample collection. 4/5 cells for day 5 samples collection. Continue compounds treatment at concentrations of 0, 0.01 µM, 0.1 µM, 1 µM, 10 µM.

- Day 5: Trypsinize cells, rinse cell pellets once with PBS, and store cell pellets at -80°C.
- Day 7: Change medium of Day 10 sample dishes, and continue compounds treatment.
- Day 10: Trypsinize cells, rinse cell pellets once with PBS once, and store cell pellets at -80°C.

  ***Each treatment condition is triplicated.**

IV. CRISPR screen (workflow shown in Supplementary Figure 7)

- Prepare sgRNA lentiviruses for targets of interest, store viruses at -80°C. Biological titer is determined by infecting 293T cells with serial diluted viral stock. The typical viral titer should be around $5x10^6$/ml.
- Day -1: Thaw cells from frozen subpools. Seed 5 $x10^5$ cells/well with 3 ml medium in 6-well plate. **Cell number estimation: total 400 lines, ~1250 cells/line.**
- Day 0: Infect cells with 2 ml medium containing 2.5 $x10^6$ viral particles and 4 µg/ml Polybrene. **This makes multiplicity of infection (MOI) to ~5.**
- Day 1: Change to fresh medium, and start Puromycin selection at 2 µg/ml.
- Day 3: Collect Day 3 samples. Split cells from each well to two 100 mm dishes: 1/5 cells for Day 13 samples collection, 4/5 cells for Day 7 samples collection. All cells are cultured in medium with 2 µg/ml Puromycin.
- Day 7: Trypsinize cells, rinse cell pellets once with PBS, and store cell pellets at -80°C.
- Day 13: Trypsinize cells, rinse cell pellets once with PBS, and store cell pellets at -80°C.

  ***Each treatment condition is triplicated.**

V. Extracting Genomic DNA

- Extract genomic DNA using QiaAmp Fast DNA Tissue kit (Qiagen), with RNase A treatment upon cell lysis to eliminate cellular RNA.
- Measure genomic DNA concentration with TapeStation 4200 (Agilent, Genomic DNA Tape).

VI. NGS library preparation and sequencing

PCR1-Amplify barcodes:

- This step is to amplify barcodes from each genomic DNA sample.

Reagents	Volume (µl)	Notes
1 µg genomic DNA	20	
Forward Primer (10 µM)	3	5'-ACAACAAGCACCGGGATAAG-3'
Reverse Primer (10 µM)	3	5'-AGGAACTGCTTCCTTCAC GA-3'
10 mM dNTP (10 mM)	2	Takara Bio. Cat#639242
10 x Titanium Taq Buffer	10	
Titanium Taq Polymerase	2	
H_2O	60	
Total	100	

PCR Conditions

94°C, 2 minutes	
94°C, 45 seconds	Total 25 cycles
65°C, 15 seconds	
72°C, 30 seconds	
72°C, 2 minutes	

- Clean PCR1 products with SPRI bead method (Beckman Coulter SRPI Select Reagent, Cat#B23317). Elute in 30 µl Qiagen EB buffer.
- Quantify eluted PCR1 product on TapeStation 4200.

<u>PCR2-NGS library:</u>

- This step is to multiplex samples.

**Reagents**	**Volume (µl)**	**Notes**
1 ng PCR1 product	20	
TruSeq-D50 Barcode F Primer (10 µM)	3	5'-AATGATACGGCGACCACCGAGATCTACAC-*Barcode*-CACGGATCGACCTGTCTCAG-3'
TruSeq-D50 Barcode R Primer (10 µM)	3	5'-CAAGCAGAAGACGGCATACGAGAT-*barcode*-GTGACTGGAGTTCAGACGTGTGCTCTTCCGATCTGCCAAAAGACGGCAATATG-3'
10 mM dNTP (10 mM)	2	Takara Bio. Cat#639242
10 x Titanium Taq Buffer	10	
Titanium Taq Polymerase	2	
H_2O	60	
Total	100	

PCR2 Conditions

94°C, 2 minutes	
94°C, 45 seconds	Total 6 cycles
65°C, 15 seconds	
72°C, 30 seconds	
72°C, 2 minutes	
94°C, 2 minutes	
94°C, 45 seconds	Total 6 cycles
71°C, 15 seconds	
72°C, 30 seconds	
72°C, 2 minutes	

- Clean PCR2 products with SPRI bead method (Beckman Coulter SRPI Select Reagent, Cat#B23317). Elute in 30 µl Qiagen EB buffer.
- Quantify eluted PCR2 product on TapeStation 4200.

NGS library sequencing:

- Mix up to 96 PCR2 products (10 nmol each) to make a sequencing pool.
- Assess the pooled sequencing library for quality and quantity with TapeStation 4200.
- Add 10% molar ratio spike-in PhiX to each pool to enhance sequence diversity.
- Submit libraries for NGS sequencing.

VII. Data analysis

- Raw NGS sequencing data was decomplexed using TruSeq-D50 Barcode pairs.
- The MAGeCK pipeline (version 0.5.9) was then used to process and analyze the 26-bp PRISM barcodes. First, the command 'mageck count' was used to map raw sequencing reads with no mismatch allowed, to the PRISM barcode library. We also conducted a thorough examination of the data quality (total reads generated per sample, percentage of mapped reads, zero cell line count, etc). The criteria for samples to pass the quality control include: mapping rate >= 70%, zero cell line count <= 5% of total cell lines in the PRISM assay.
- Raw cell line counts were finally normalized to the median count of the negative control cell lines included in the PRISM cell line pool.

Compound screen

- For each treatment condition, raw count of each tumor cell line was normalized to two 293T control lines (assuming 293T does not respond to compound treatment). **For those compounds that have effect on 293T cells, raw counts were further normalized according to 293T DRC curve obtained from CTG assay.**
- Normalized count of each compound treatment condition was then divided by count of DMSO treated samples, and AUC was calculated to determine the sensitivity of cell lines to this compound.

CRISPR screen

- For each treatment condition, raw count of each tumor cell line was normalized to two 293T-Cas9del control lines (These cells express truncated Cas9 protein so that they don't respond to sgRNA-mediated knockout).
- Normalized count of each sgRNA treatment condition was then divided by count of Non-targeting sgRNA treated samples, and relative depletion ratio was calculated to determine the sensitivity of cell lines to this genetic knockout.

Cell line	EGFR mutation	Other mutation	EGFR expression	EGFR copy number
HCC827	p.ELREA701del	TP53	8.984609855	3.2468
PC-9	p.ELREA746del	TP53	6.400663841	NA
HCC4006	p.ELR746del	TP53	5.805899116	1.3014
NCI-H1650	p.ELREA701del	TP53, PTEN?	5.363228432	1.1195
NCI-H292	WT		5.360771013	0.5242
SK-MES-1	WT	TP53	5.22129564	0.5968
HCC44	WT	TP53, KRAS	5.11268814	0.3605
A549	WT	KRAS	4.772872702	0.1163
NCI-H1975	L858R, T790M	TP53, PIK3CA, CDKN2A	4.722315693	0.793
NCI-H2126	WT		4.454446149	0.3287
NCI-H1755	WT	TP53, BRAF	4.349176193	0.4782
NCI-H441	WT	TP53, KRAS	4.000894033	-0.2181
NCI-H2009	WT	TP53, KRAS	3.647897428	-0.0483
NCI-H358	WT	KRAS	3.286860177	-0.118
NCI-H23	WT	TP53, KRAS	3.282020555	0.4
NCI-H1299	WT	NRAS	2.795867648	-0.2912
NCI-H460	WT	KRAS, PIK3CA	2.290724803	-1.3464
NCI-H2170	WT	P53	0.424679019	0.3615
NCI-H520	WT	P53	-2.47360452	0.0852
NCI-H522	WT	MTOR	-3.840191935	0.8515

Supplementary Table 1. Characteristics of 20 NSCLC cell lines in the pilot EGFR screen.

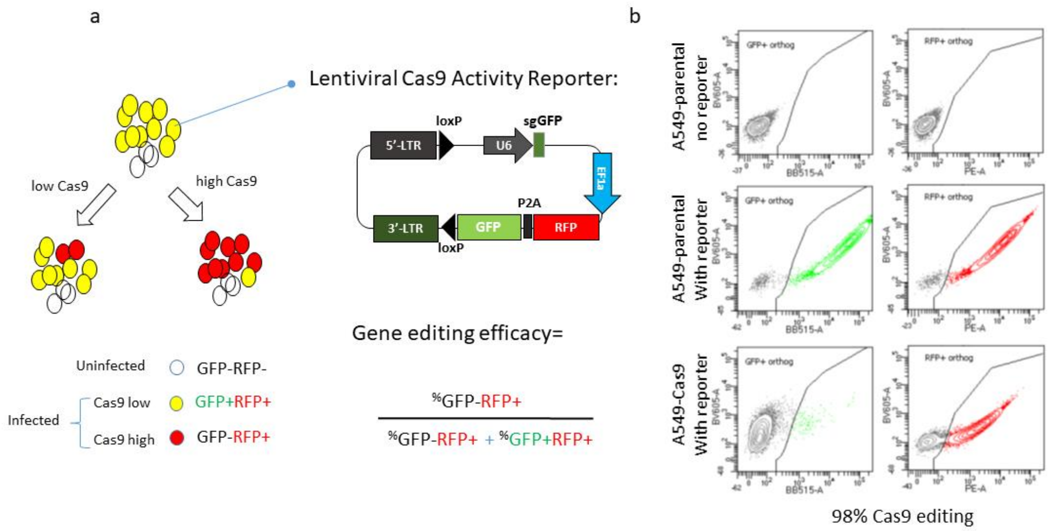

Supplementary Figure 1. Lentiviral Cas9 activity reporter assay for CRISPR editing efficiency quality control. a. The lentiviral vector expresses RFP-P2A-GFP and sgRNA against GFP. When it infects a cell with decent Cas9 activity, the sgRNA edits GFP ORF and silences its expression, thus the cell only has RFP signal. The Cas9 editing efficacy is calculated as indicated. **b.** An example of flow cytometry result from A549 parental and Cas9-engineered cells when infected with lentiviral Cas9 activity reporter.

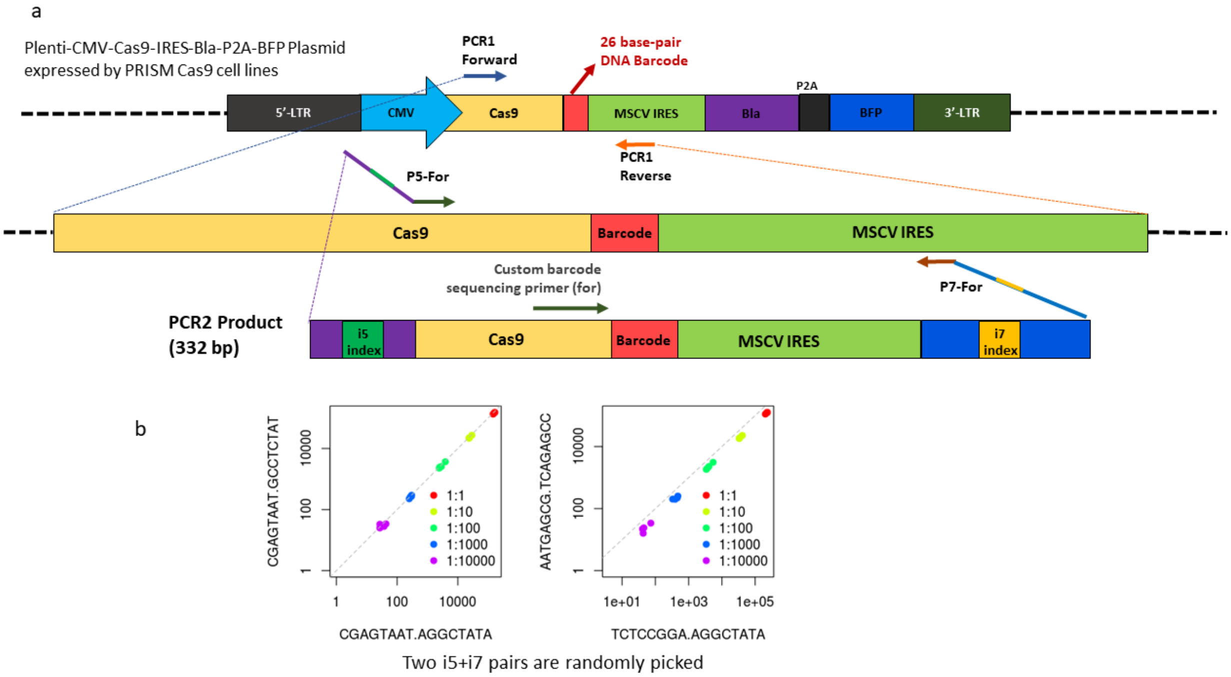

Supplementary Figure 2. Next-generation sequencing library preparation for detecting relative barcode abundance in the sample mix. a. Diagram of two-round PCR amplification of barcodes. PCR1 uses universal primers to amplify barcode regions from genomic DNA or plasmids mix.PCR2 adds dual-indexed Illumina primers to the barcode libraries for multiplexed sequencing. **b.** 20 barcode plasmids were mixed at different concentrations to evaluate the detection limit of Next-generation sequencing protocol.

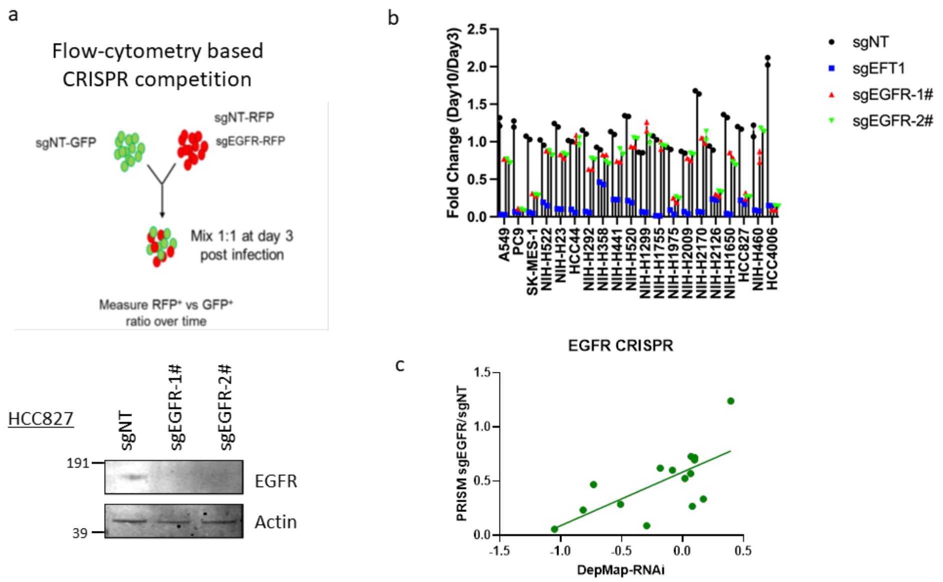

Supplementary Figure 3. EGFR dependency was evaluated in 20 NSCLC cell lines using individual CRISPR competition assay. a. Diagram showing the principle of CRISPR competition assay. **b.** 7-day CRISPR competition assay results of the 20 NSCLC cell lines after EGFR knockout. sgNT, non-targeting sgRNA used as negative control; sgETF1, sgRNA against essential gene ETF1 used as positive control; sgEGFR-1# and -2#, two sgRNAs against EGFR gene. Error bars represent standard error of the mean. n=2 biologically independent samples. **c.** Correlation analysis of EGFR CRISPR PRISM assay vs DepMap gene effect score (DEMETER, 15 cell lines, r=0.6813). Related to **Figure 2f**.

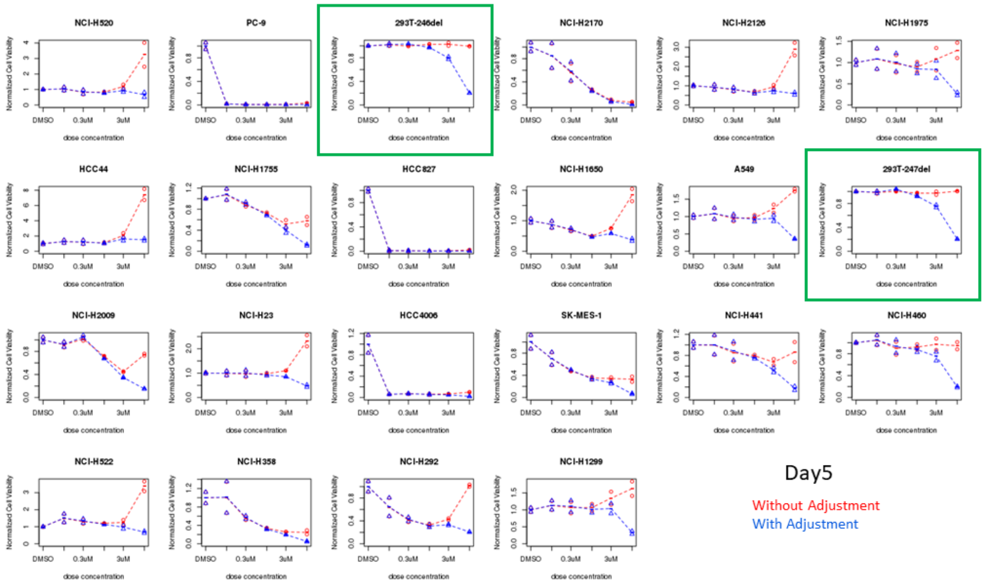

Supplementary Figure 4. Adjustment of Dose responsive curve after Erlotinib treatment. The adjustment was performed according to individual 293T CTG assay result. Detailed methodology was described in *Supplementary methods*.

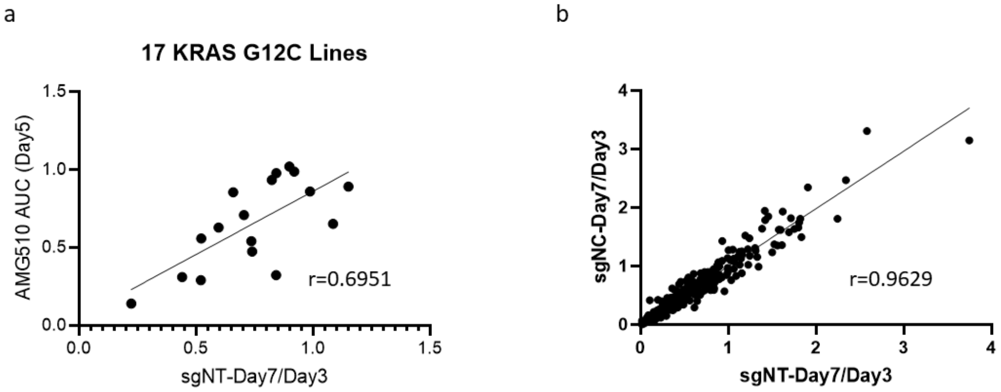

Supplementary Figure 5. Related to Figure 3. a. Correlation of KRAS CRISPR knockout vs AMG-510 treatment of 17 KRAS G12C cancer cell lines in the BMS-PRISM screen. b. Relative cell abundance of each cell line in the BMS-PRISM upon the infection of sgNT and sgNC.

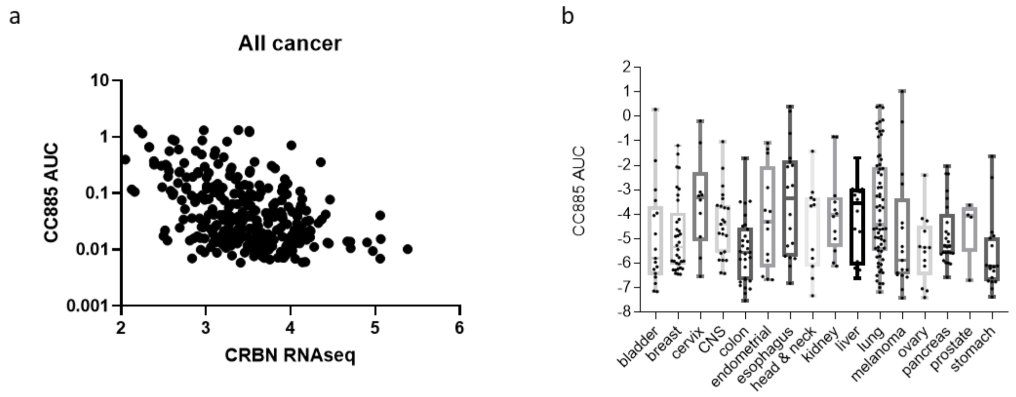

Supplementary Figure 6. CC-885 PRISM screen result indicated the relation between cytotoxicity and Cereblon expression level. a. Correlation of CRBN mRNA score (Cancer Cell Line Encyclopedia, mRNA expression RNAseq) and 5-day CC-885 AUC in PRISM screen. b. CC-885 AUC distribution across major cancer indications in BSM-PRISM library. Error bars represent standard error of the mean. Cell line numbers in each group were: bladder (n=17), breast (n=34), cervix (n=10), CNS (n=23), colon (n=29), endometrial (n=13), esophagus (n=18), head & neck (n=11), kidney (n=12), liver (n=19), lung (n=65), melanoma (n=17), ovary (n=13), pancreas (n=25), prostate (n=4), stomach (n=18).

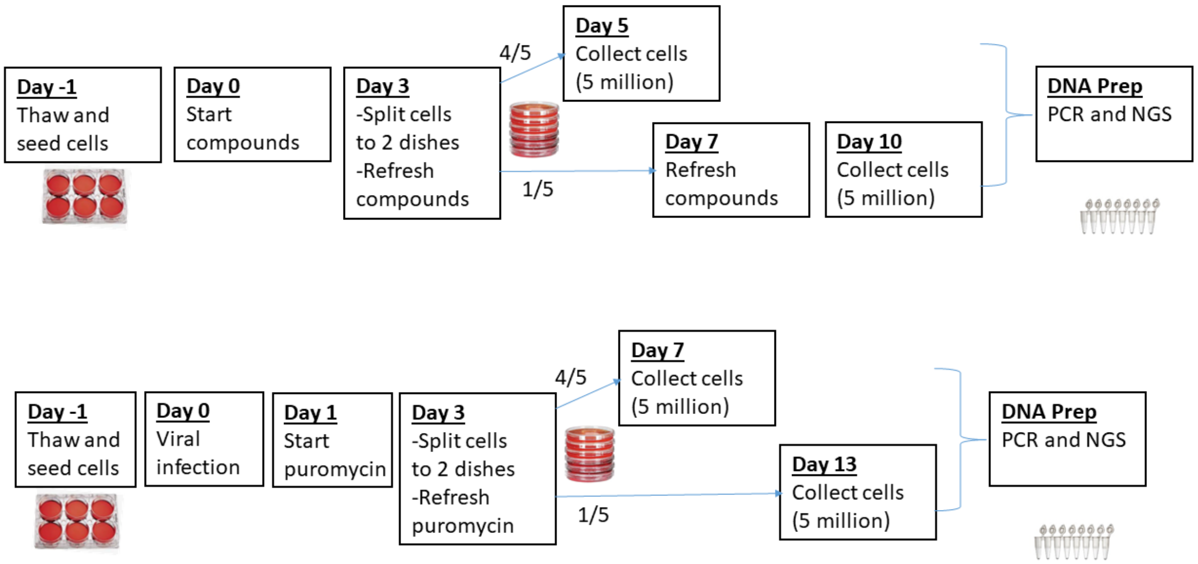

Supplementary Figure 7. Workflow of BMS-PRISM screen. Detailed methodology is described in *Supplementary Methods*.

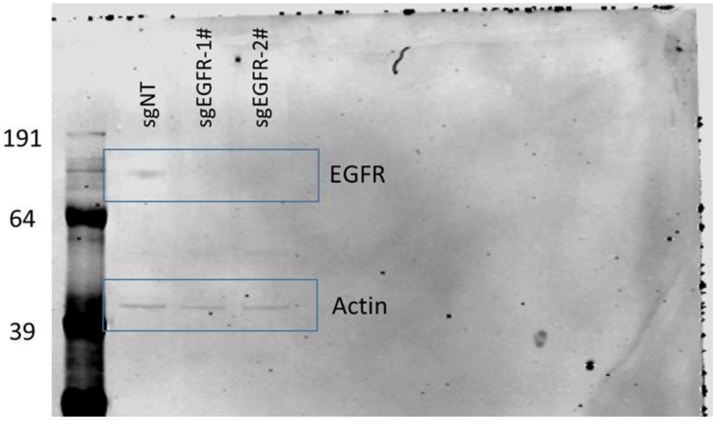

Supplementary Figure 8. Full scan of western blot in Supplementary Figure 3a.
